# Coats' disease: An Indian perspective

**DOI:** 10.4103/0301-4738.60081

**Published:** 2010

**Authors:** Pukhraj Rishi, Ekta Rishi, Mahesh Uparkar, Tarun Sharma, Lingam Gopal, Pramod Bhende, Muna Bhende, Pratik Ranjan Sen, Parveen Sen

**Affiliations:** Shri Bhagwan Mahavir Vitreoretinal Services, Sankara Nethralaya, 18, College Road, Chennai - 600 006, India

**Keywords:** Children, Coats' disease, cryotherapy, laser photocoagulation, macular edema, retinal detachment, retinal telangiectasia

## Abstract

**Aim:**

To describe the clinical features, treatment and outcome patterns in 307 eyes with Coats' disease.

**Materials and Methods:**

Retrospective chart review of patients diagnosed with Coats' disease between January 1996 and January 2006 from a single referral center in southern India.

**Results:**

Two hundred and eighty patients (307 eyes) with mean age of 15.67 years (range: Four months-80 years) were included. Decreased vision (77%), unilateral affection (90%) and male preponderance (83.4%) were chief presenting features. Anterior segment involvement was seen in 67 (21.8%) eyes. Retinal telangiectasia were seen in 302 (99%) eyes, exudation in 274 (89%) eyes and retinal detachment in 158 (51.5%) eyes. Four-quadrant disease was seen in 207 (67.2 %) eyes. Visual acuity was < 20/200 in 249 (80.9%) eyes. One hundred and nine of 176 treated eyes (61.93%) had favorable anatomical outcome; 207 of 280 eyes (74%) had an optimal structural outcome. Seventeen (5.3%) eyes were enucleated. Complications following treatment included *phthisis bulbi* (7%), neovascular glaucoma (5%), epiretinal membrane (4.4%) and rubeosis iridis (4.4%).

**Conclusion:**

Indian patients with Coats' disease have a high male predominance, the majority of whom present with severe visual impairment and extensive four-quadrant exudation. Unusual presentations such as pain, vitreous hemorrhage and a high incidence of anterior segment involvement are distinctive to Indian eyes.

George Coats in 1908 described a conundrum of ocular findings such as unilateral retinal vascular abnormalities and retinal exudation that usually occurred in young males.[1] Leber, in 1912, reported a condition characterized by multiple retinal aneurysms associated with retinal degeneration that was seen in young males.[2] These conditions were recognized eponymously for almost half a century as two separate entities, until Reese in 1955, elucidated the similarities between Coats' disease and Leber's miliary aneurysms.[3] Retinoblastoma, an important ocular tumor of childhood forms a major differential diagnosis of this disease, and considering its significance, becomes even more imperative for the treating physician to make an accurate diagnosis. Coats' disease more commonly affects children and has a clear male predilection and a unilateral affection.[4–6] Shields *et al*. in an assertive account of their experience over 24 years showed the natural course of the disease to be one marked with recurrences.[7] While most of the current reports on Coats' disease have emanated from the Western world, there have hardly been any detailed accounts from the Indian subcontinent.[4–16] This study attempts to fill in the lacuna in the knowledge about this condition in the Indian/ south Asian context.

## Materials and Methods

This study was a retrospective case series which enlisted patients diagnosed with Coats' disease between January 1996 and January 2006. Coats' disease was diagnosed if an eye had:

- Idiopathic, retinal telangiectasia
- With or without intra or subretinal exudation and
- Typically seen unilaterally in young males.

Retinal telangiectasia were defined as dilated, irregular caliber, small to medium-sized retinal vessels; exudative retinopathy as yellow exudation in the sensory retina or subretinal space. Other causes of vasculopathy with evident exudation were excluded. Patient details included age, sex, laterality, duration of symptoms and other vital history. Visual acuity (Snellen's Chart at 6 m), intraocular pressure and anterior segment findings were noted. A thorough fundus examination with binocular indirect ophthalmoscope and a detailed fundus drawing aided with fundus photographs were utilized to enter pertinent data. The disease extent was classified into three zones, the macula (Clinical macula: Area between the temporal vascular arcades) being Zone 1; the mid-periphery, between the retinal vascular arcades and equator being Zone 2 and the peripheral fundus anterior to this being Zone 3. Staging of the disease was done based on the extent and the quadrant(s) of retina involved.[12] Treatment was indicated in cases of visibly leaking vascular malformations, exudation at fovea, exudative retinal detachment and end-stage complications like neovascular glaucoma. The nature of treatment and the retinal and visual outcomes at the last visit were recorded. The frequency of complications such as complicated cataract, rubeosis iridis, neovascular glaucoma, anterior chamber cholesterolosis and other posterior segment complications was determined. Fundus photography and fluorescein angiography were done, whenever indicated and feasible. Imaging with ultrasound and computed tomography (CT) scan were primarily performed to exclude retinoblastoma or where media haze precluded fundal view. Management options included observation alone, laser photocoagulation, cryotherapy, scleral buckling, vitreous surgery, external subretinal fluid drainage and enucleation. Optimal structural outcome was defined as cessation of telangiectatic activity with preservation of globe shape without neovascular glaucoma. Favorable structural outcome was defined as an attached retina besides the features of an optimal structural outcome. Favorable functional outcome was defined as improved vision, better than counting fingers. Histopathologic examination was done in all enucleated eyes for a tissue-based diagnosis. Cytopathologic and biochemical confirmation of subretinal fluid obtained after the external drainage procedure was done. Statistical analysis was done using Univariate and Multivariate analysis for factors predicting a favorable structural and functional outcome. Parametric test of significance (paired *t* test) was used for continuous data while non-parametric test (Kruskal Wallis test) was used for non-parametric data. The SPSS Version 9 (SPSS Inc, Chicago, USA) was used for analysis.

## Results

Over the last 10 years, 280 patients were diagnosed with Coats' disease. The mean age at presentation was 15.67 + 11.6 years (range: Four months to 80 years) with a median age of 11 years. Of the 280 patients, 251 (90%) cases were unilateral and 28 (10%) were bilateral. Males constituted a majority; 256 eyes (83.4%) belonged to male patients, while 51 (17.2%) eyes were that of females. The average duration of complaints was 4.2 months (Range: Four days to six years). The most common clinical symptom at presentation was decrease in visual acuity, seen in 221 (77%) eyes followed by ocular deviation in 69 (22.4%) eyes, pupillary white reflex (leucocoria) in 62 (20.6%) eyes and ocular pain in nine (2.9%) eyes while five (1.6%) eyes were asymptomatic. The distribution of visual acuity recorded at presentation was thus: 20/40 or better in 42 (13.6%) eyes, less than 20/40 and better than 20/120 in 17 (5.5%) eyes, less than 120 and better than 20/2000 in 62 (20.1%) eyes and 186 (60.8%) eyes had hand motion to nil perception of light.

All eyes were graded according to the stage of the disease at time of diagnosis [Table 1]. Of the 67 eyes (22%) with anterior segment findings [Table 2], raised intraocular pressure (>21 mmHg, Goldmann applanation) was noted in 19 eyes (6%). Retinal telangiectasia were seen in 302 eyes (99%). Exudation (intra/sub-retinal) was seen in 274 eyes (89%). Retinal detachment was noted in 153 eyes (50%). Of these, total retinal detachment was seen in 98 (32%) eyes, while subtotal (>3 quadrants and <4 quadrants) detachment was noted in 55 eyes (18%). Retinal macrocysts were seen in 12 (4%) eyes indicating the long duration of the detachment. Vitreous hemorrhage at presentation precluded a complete posterior segment evaluation in 14 eyes (4.3%). Disc neovascularization was noted in seven (2.3%) eyes and macular hole in one (0.3%) eye.

**Table 1:** Staging of eyes with Coats' disease, at presentation

Stage	Zone I (%)	Zone II (%)	Zone III (%)	Total (%)
1	2	2	1	5 (1.6)
2A	4	17	17	38 (12.4)
2B	30	7	48	85 (27.7)
3A1	3	10	13	26 (8.5)
3A2	3	6	16	25 (8.1)
3B	6	9	84	99 (32.2)
4	1	0	25	26 (8.5)
5	0	0	3	3 (1.0)
	49 (16.0)	51 (16.6)	207 (67.4)	307

**Table 2:** Anterior segment findings in the 307 eyes, at presentation

Anterior segment findings*	N	Percentage
Normal	240	78.2
Cataract	17	5.5
Neovascularization of the iris	17	5.5
Anterior chamber cells/flare	13	4.1
Anterior vitreous cells	7	2.4
Shallow anterior chamber	12	4
Raised IOP	19	6
Anterior chamber cholesterolosis	4	1.1
Corneal edema	8	2.5

* Many patients had a combination of signs hence number of eyes may be more than 307

Ultrasound scan was done in 70 eyes (22.7%) while fluorescein angiography was done in 128 eyes (41.5%). A CT scan was required as an additional investigation in four eyes (1.2%) to rule out retinoblastoma. Cytopathologic and biochemical evaluation of the subretinal fluid in 34 eyes (11%) was consistent with the diagnosis of Coats' disease. Eosinophilic foamy histiocytes with cholesterol crystals were typically seen in all 34 samples.

Observation was adjudged appropriate in 163 eyes (53%) while 103 (33.3%) eyes underwent laser photocoagulation.[17] Thirty eyes (9.8%) underwent scleral buckling, 13 eyes (4.2%) had vitrectomy and 24 eyes (7.8%) were treated with cryotherapy.[18–21] Twenty-one eyes (6.8%) with advanced disease underwent external drainage of sub-retinal fluid.[22] Seventeen (5.3%) eyes underwent enucleation while three patients did not undergo treatment. The outcome of management in the 289 eyes that had a follow-up is shown in Table 3. Complications following treatment included: *Phthisis bulbi* in 22 (7%) eyes, glaucoma in 16 (5%) eyes, hypotony in 14 (4.5%) eyes, neovascularization of iris in 13 (4.4%) eyes, epiretinal membrane in 13 (4.4%) eyes, vitreous hemorrhage in 12 (4.2%) eyes and iatrogenic retinal break causing rhegmatogenous retinal detachment in three (1%) eyes.

**Table 3:** Outcome of treatment in eyes with follow-up

Management options	N (%)	Retina (attached*/detached)	OR	RR	*P* value
Observation only+	158	39/119			
Laser	80 (33.3)	56/24	7.11	2.51	0.001
Scleral buckle	18 (9.8)	6/12	1.52	1.12	>0.05
Cryotherapy	17 (7.8)	11/6	5.59	2.34	>0.05
Subretinal fluid drainage	21 (6.8)	12/9	4.06	1.74	0.004
Enucleation	15 (5.3)	Not applicable			
Vitrectomy	8 (4.2)	7/1	21.06	6.03	>0.05

OR - Odds ratio, RR - Relative ratio;

* Attached retina is considered as reference, the risk of detachment is determined.

+ Observation group was considered reference when determining odds ratio. N.B - Many eyes had combination of treatment; numbers may not add up to 289

In the analysis of structural outcome, we analyzed patients with at least 12 months of follow-up from presentation. Among those meeting this criterion, 116 eyes (93 patients) were found to have an average follow-up of 14.2 months (range 12-44 months). Univariate analysis [Table 4a] of factors responsible for an attached posterior pole retina at last visit showed attached retina at first visit, treatment with scleral buckling and peripheral disease to be statistically significantly associated with detached retina at last visit and a correspondingly poor visual outcome. Multivariate analysis [Table 4b] showed even more significance of these factors in the final retinal outcome; retina status at first visit (*P* = 0.008), peripheral disease (*P* = 0.024) and treatment with scleral buckling (*P* = 0.028).

**Table 4a:** Univariate analysis for anatomical outcome at final visit in 116 eyes with follow-up of one year or more

	Retinal outcome		Odds ratio	95% CI	*P* value
				
	Improved Worsened				
Baseline retinal					
status					
Attached	19	45	1.407	0.608-3.256	0.424
Detached	12	40			
Observation					
No	16	51	0.711	0.311-1.626	0.418
Yes	15	34			
Laser				
No	20	40	2.045	0.874-4.786	0.096
Yes	11	45			
Vitrectomy					
No	29	78	1.407	0.608-3.256	0.424
Yes	2	7			
Encirclage					
No	29	65	4.462	0.978-20.359	0.038
Yes	2	20			
Cryotherapy					
No	26	78	0.467	0.136-1.597	0.217
Yes	5	7			
Quadrant					
2 or less	11	19	1.911	0.780-4.677	0.153
More than 2	20	66			
Zone					
Peripheral	21	75	0.280	0.103-0.762	0.010
Central	10	10			
Retinal status at first follow-up					
Attached	14	57	0.405	0.175-0.937	0.032
Detached	17	28			

CI - Confidence interval

**Table 4b:** Multivariate analysis for anatomical outcome at final visit in 116 eyes with follow-up of one year or more

	Retinal outcome		Odds ratio	95% CI	*P* value
				
	Improved Worsened				
Baseline retinal					
status					
Attached	19	45	1.289	0.432-3.845	0.649
Detached	12	40			
Observation				
No	16	51	7.017	0.657-74.944	0.107
Yes	15	34			
Laser					
No	20	40	12.176	1.198-123.726	0.035
Yes	11	45			
Vitrectomy					
No	29	78	1.277	0.168-9.715	0.813
Yes	2	7			
Encirclage					
No	29	65	16.364	1.351-198.182	0.028
Yes	2	20			
Cryotherapy					
No	26	78	0.784	0.097-6.350	0.820
Yes	5	7			
Quadrant					
2 or less	11	19	0.708	0.207-2.427	0.583
More than 2	20	66			
Zone					
Peripheral	21	75	0.263	0.083-0.841	0.024
Central	10	10			
Retinal status at first follow-up					
Attached	14	57	0.266	0.099-0.712	0.008
Detached	17	28			

CI - Confidence interval

**Figure 1:**
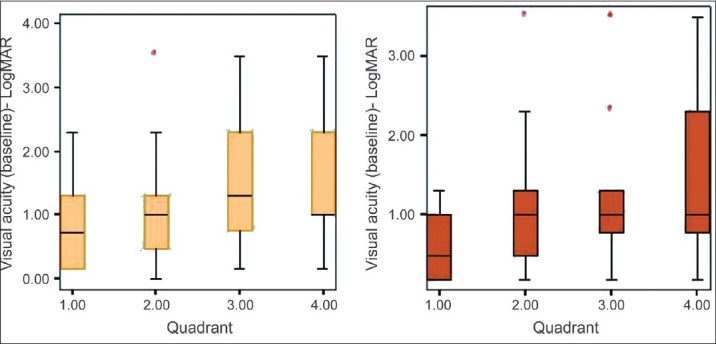
Comparative graphs showing the change in baseline visual acuity compared with disease in each quadrant of retina affected

The visual acuity in the eyes with one-year follow-up did not show any significant change in the final visual acuity (mean logMAR 1. 74 baseline and 1.75 at last visit in 116 eyes) *P* = 0.794, paired *t* test. Also, there was no statistical difference between the baseline and final visual acuity according to quadrantic disease distribution [Fig. 1].

## Discussion

**Figure 2:**
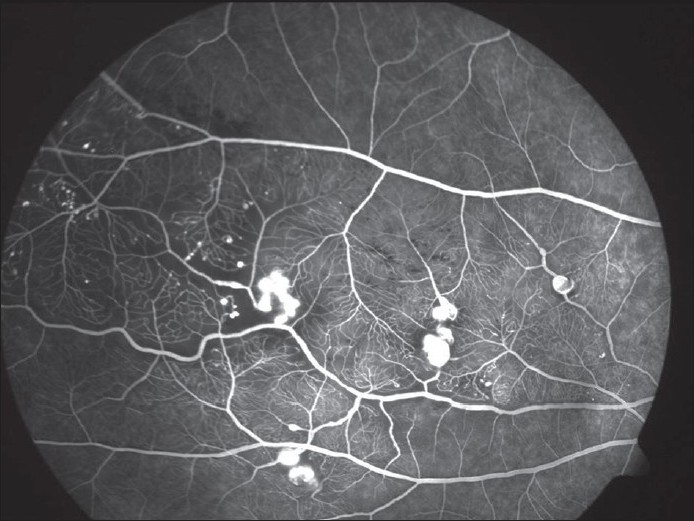
Fluorescein angiogram reveals several areas of hyperfluorescence from leaking retinal telangiectasia, giving a typical ‘light bulb’ appearance

**Figure 3:**
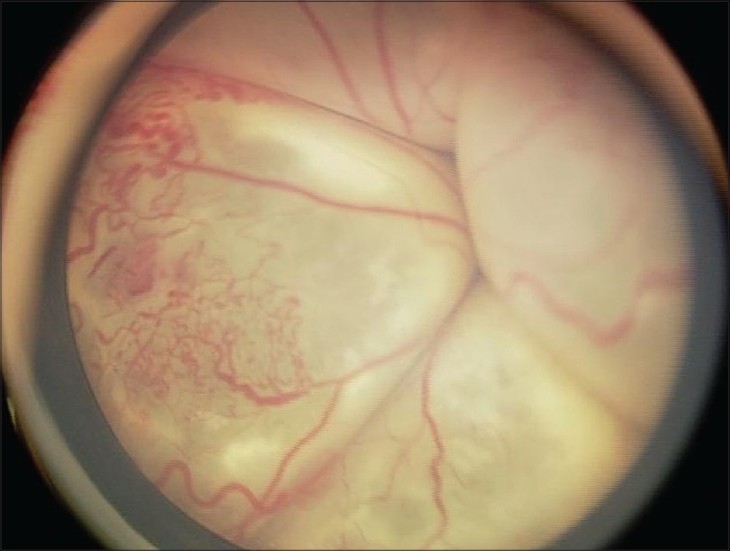
Fundus photograph revealing a bullous, total retinal detachment. Noteworthy retinal features are retinal vessel tortuosity, irregular vessel caliber, aneurismal dilatations and subretinal exudation

**Figure 4:**
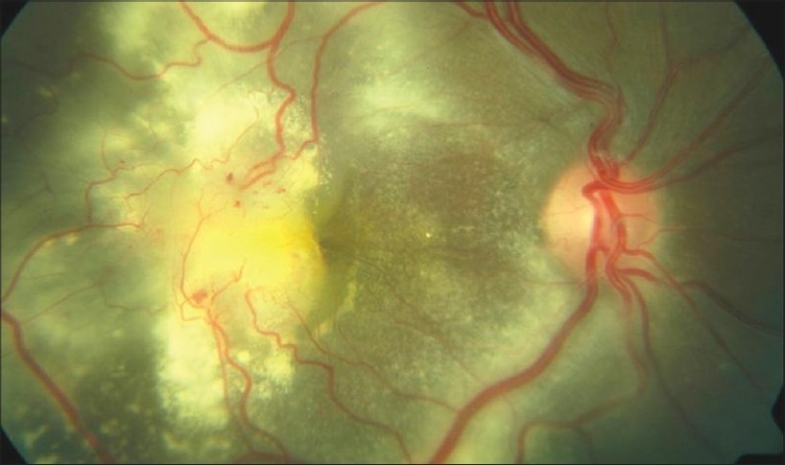
Color fundus photograph revealing macular details—several macular telangiectasia, submacular fluid and exudates are also noted

**Figure 5:**
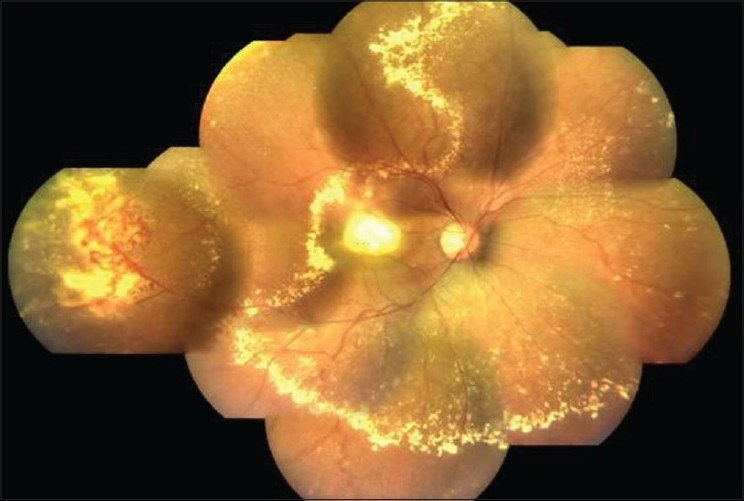
Color fundus montage reveals a cluster of retinal telan giectasia in the temporal periphery. A row of retinal exudates in the mid-periphery and dense macular exudation are also made out

**Figure 6:**
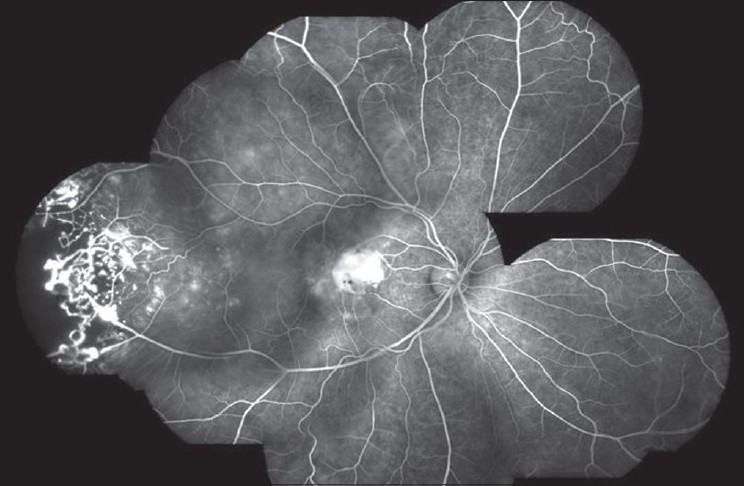
A montage image of fluorescein angiogram of the same eye as in Figure 4. Leaking retinal telangiectasia are highlighted and an adjacent area of capillary non-perfusion is made out

**Figure 7:**
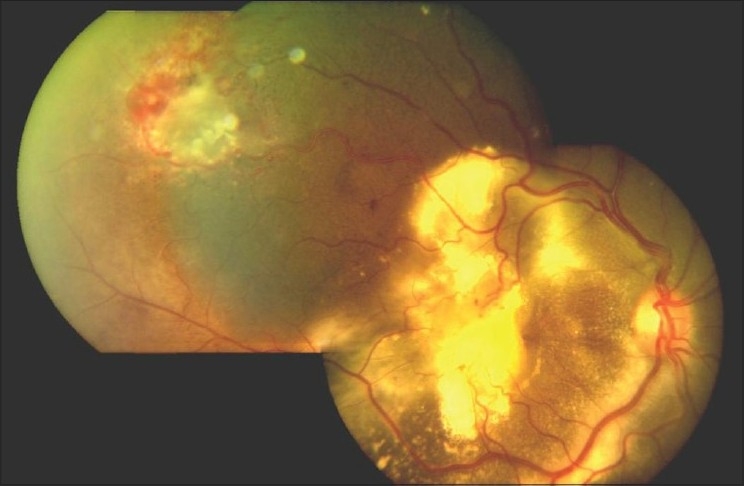
Color fundus montage reveals the presence of both peripheral and macular retinal telangiectasia. Exudative retinal detachment and dense macular exudation are noteworthy features

**Figure 8:**
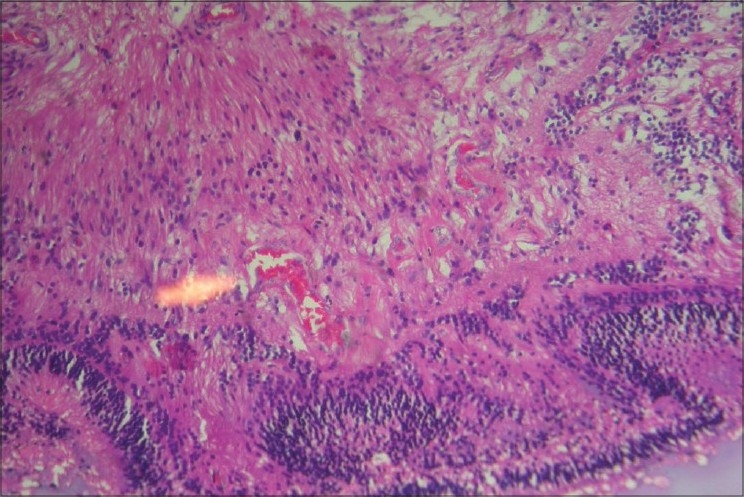
Photomicrograph showing retinal telangiectatic vessels (arrow) (H and E stain, ×100)

The hallmark lesion of this disease is idiopathic retinal telangiectasia which shows up as ‘light bulb appearance’ on fluorescein angiography [Fig. 2]. Other features of this disease include irregular retinal vessel dilatations, retinal vessel tortuosity, areas of capillary non-perfusion, intra- and/or subretinal exudation and retinal detachment [Fig. 3]. Macular exudation may be caused by macular telangiectasia [Fig. 4], peripheral retinal telangiectasia [Figs. 5 and 6] or both [Figs. 7 and 8] elucidates histopathological study of such an eye revealing intraretinal telangiectasia. Coats' disease must be differentiated from several other clinical entities which simulate its fundus picture, especially in children such as retinoblastoma, toxocariasis, persistent fetal vasculature syndrome, retinitis pigmentosa with Coats-like reaction and diseases with vascular changes at the vitreoretinal interface (retinopathy of prematurity, familial exudative vitreoretinopathy, incontinentia pigmenti).

In order to unravel possible differences in the disease manifestation as seen in this part of the world to that reported in the predominantly Western literature available, we compared our findings with several other studies, particularly the one by Shields *et al*.[7] Anterior segment abnormalities in Coats' disease have been infrequently reported. The incidence of cataract and rubeosis iridis was 8% each in the studies by Shields *et al*.,[7] as compared to 5.5% each in our study. Amongst posterior segment findings, a larger percentage of eyes in our study had more than one-quadrant exudation (91.6% versus 73%) or even four-quadrant exudation (67.2% versus 55%) at presentation. However, exudative retinal detachment (50% versus 80%) and retinal macrocysts (4% versus 11%) were seen much less often. We found a bimodal pattern of onset, 31 patients (11%) being more than 35 years of age at presentation with mean age being 46.72 years (range 35 to 80 years). Male predominance (74.19%) and bilaterality of disease (6.46%) were less marked than the overall group. The adult-onset disease is more localized, slowly progressive and amenable to treatment with a greatly reduced incidence of neovascular complications.[23,24] Nucci *et al*. have reported anatomical benefits from treatment with a yellow-dye laser.[25] We have not seen any patient with a positive family history for Coats' disease.

Of the 15 eyes of preschool children (<four years) in our study, nine eyes had retinal detachment, while seven eyes had exudation >three quadrants. Stage 3A1 or greater in younger children usually has a poor prognosis.[7,13]

Twenty-one eyes with advanced Coats' disease were managed with external subretinal fluid drainage and cryo therapy. Retinal reattachment was achieved in 57% eyes while anatomical stabilization was achieved in 85.7% eyes (mean follow-up: 15 months).

We compared the causes of poor visual outcome in the treated eyes with those reported by Shields *et al*.[13] and found that subfoveal fluid (21.8% versus 47%), subfoveal fibrosis (26.4% versus 29%) and foveal exudation (16.95 versus 11%) were the three commonest causes in both the studies, albeit with some variations. The incidence of epiretinal membrane (4.4% versus 2.5%) and macular edema (11% versus 7.5%) was comparatively higher in our study. In the final outcomes, anatomical stabilization achieved was comparable (74% versus 76%) while 40% patients in the present study as compared to the 20% reported in the quoted study had a final visual acuity of more than 20/200. Seventeen (5.3%) eyes were eventually enucleated in this series, whereas 20 (16%) eyes had to be removed in the quoted study.

On an average, Indian patients present at least five years later than their Western counterparts (mean age: 16.5 years versus 11 years) with an average duration of symptoms of 2.5 + 2.8 years. While more than half (55.5% versus 43%) the patients present with diminution of vision, a large majority (80.9% versus 76%) had visual acuity worse than 20/200. Male predominance is even more pronounced (83.2% versus 76%). Bilateral disease is seen twice (10% versus 5%) more commonly. There appears to be a bimodal presentation, the first major peak at a mean age of 16.5 years and the second smaller peak at a mean age of 46.7 years. Anterior segment involvement is more than twice (21.8% versus 10%) more common. Macular telangiectasia is seen five times (5.5% versus 1%) more commonly. Vitreous hemorrhage is a peculiar presenting feature. Almost half (49.2%) the eyes had retinal detachment at presentation, while more than three-fourths of eyes (78.3%) had disease involvement of three quadrants of fundus or more. Extensive four-quadrant retinal exudation is also seen more frequently (67.2% versus 55%). However, exudative (secondary) retinal detachment and retinal macrocysts are seen much less often. Unusual presentations such as pain, vitreous hemorrhage and increased incidence of anterior segment involvement are peculiar to Indian eyes. Treatment in the early stages is often successful in achieving anatomical stabilization. However, advanced cases are difficult to treat and results are disappointing. Visual improvement in classic Coats' disease of childhood onset is usually very minimal and frequently, complications may compromise visual function despite an attached retina.

## References

[1] Coats G (1908). Forms of retinal disease with massive exudation. R Lond Ophthalmol Hosp Rep.

[2] Leber T (1912). Über eine durch Vorkommen multipler Miliaraneurysmen charakterisierte Form von Retinaldegeneration. Albrecht von Grafes Archiv für Ophthalmologie.

[3] Reese AB (1956). Telangiectasias of the retina and Coats' disease. Am J Ophthalmol.

[4] Morales AG (1965). Coats' disease. Natural history and results of treatment. Am J Ophthalmol.

[5] Manschott WA, DeBruijn WC (1967). Coats' disease: Definition and pathogenesis. Br J Ophthalmol.

[6] Chang M, McLean IW, Merritt JC (1984). Coats' disease: A study of 62 Histologically confirmed cases. J Pedaitr Ophthalmol Strabismus.

[7] Shields JA, Shields CL, Honavar SG, DeMirci H (2001). Clinical variations and complications of Coats' disease in 150 cases: The 2000 Sanford Gifford memorial lecture. Am J Ophthalmol.

[8] Egerer I, Tasman W, Tomer TL (1974). Coats' disease. Arch Ophthalmol.

[9] Ridley ME, Shields JA, Brown GC, Tasman W (1982). Coats' disease. Evaluation of management. Ophthalmology.

[10] Haik BG (1991). Advanced Coats disease. Trans Am Ophthalmol Soc.

[11] Tarikkanen A, Laatikainen L (1983). Coats' disease: Clinical, angiographic, histopathological findings and clinical management. Br J Ophthalmol.

[12] Budning AS, Beon E, Gallie BL (1998). Visual prognosis of Coats' disease. J AAPOS.

[13] Shields JA, Shields CL, Honavar SG, Demirci H, Cater J (2001). Classification and management of Coats disease; The 2000 Proctor lecture. Am J Ophthalmol.

[14] Silodor SW, Augsburger JJ, Shields JA, Tasman W (1988). Natural history and management of advanced Coats' disease. Ophthalmic Surg.

[15] Char DB (2000). Coats' syndrome: Long term follow up. Br J Ophthalmol.

[16] Shienbaum G, Tasman W (2006). Coats' disease: A Lifetime Disease. Retina.

[17] Sneed SR, Blodi CF, Pulido JS (1989). Treatment of Coats' disease with the binocular indirect Argon laser photocoagulator. Arch Ophthalmol.

[18] Yoshizumi MO, Kreiger AE, Lewis H, Foxman B, Hakakha BA (1995). Vitrectomy techniques in late stage Coats'-like exudative retinal detachment. Doc Ophthalmol.

[19] Harris GS (1970). Coats' disease diagnosis and treatment. Can J Ophthalmol.

[20] Schmidt-Erfurth U, Lucke K (1995). Vitreoretinal surgery in advanced Coats' disease. German J Ophthalmol.

[21] Jones JA, Kroll AJ, Lou PL, Ryan EA (2001). Coats' disease. Int Ophthalmol Clin.

[22] Han ES, Choung HK, Heo JW, Kim SJ, Yu YS (2006). Effects of external subretinal fluid drainage on secondary glaucoma in Coats' disease. J AAPOS.

[23] Smithen LM, Brown GC, Brucker AJ, Yannuzzi LA, Klais CM, Spaide RF (2005). Coats' disease. diagnosed in adulthood. Ophthalmology.

[24] Black GC, Perveen R, Bonshek R, Cahill M, Clayton-Smith J, Lloyd IC (1999). Coats' disease of the retina (unilateral retinal telangiectasis) caused by somatic mutation in the NDP gene: A role for norrin in retinal angiogenesis. Hum Mol Genet.

[25] Nucci P, Bandello F, Serafino M, Wilson ME (2002). Selective photocoagulation in Coats disease: Ten-year follow-up. Eur J Ophthalmol.

